# Prognostic Factors for Clinical Response in Systemic Lupus Erythematosus Patients Treated by Allogeneic Mesenchymal Stem Cells

**DOI:** 10.1155/2019/7061408

**Published:** 2019-05-02

**Authors:** Lihui Wen, Myriam Labopin, Manuela Badoglio, Dandan Wang, Lingyun Sun, Dominique Farge-Bancel

**Affiliations:** ^1^Department of Rheumatology and Immunology, The Affiliated Drum Tower Hospital of Nanjing University Medical School, Nanjing 210008, China; ^2^Unité de Médecine Interne: Maladies Auto-Immunes et Pathologie Vasculaire (UF 04), Centre de Référence des Maladies Auto-Immunes Systémiques Rares d'Ile-de-France (Site Constitutif), Filière FAI2R, Hôpital Saint-Louis Assistance Publique Hôpitaux de Paris (AP-HP), 1, Avenue Claude-Vellefaux, 75010 Paris, France; ^3^Institut de Recherche Saint-Louis EA-3518, Université Paris Diderot, Sorbonne Paris Cité, Paris, France; ^4^EBMT Paris office, Department of Haematology, Hôpital Saint-Antoine, Paris, France; ^5^INSERM UMR S 938, Université Pierre et Marie Curie, 184, Rue du Faubourg-Saint-Antoine, Paris, France; ^6^Department of Internal Medicine, McGill University, Montreal, Canada

## Abstract

Systemic lupus erythematosus (SLE) is an autoimmune disease with a broad range of clinical manifestations and a heterogeneous disease course. There is no cure for SLE, but current standard pharmacotherapies can improve disease prognosis in most patients. However, some patients are refractory to conventional treatments and require alternative treatment options. The present study is aimed at identifying predictors of clinical response to allogeneic bone marrow-derived or umbilical cord-derived mesenchymal stem cell (BM-/UC-MSC) transplant in SLE. All adult patients identified in the Nanjing database with an SLE Disease Activity Index (SLEDAI) score ≥ 8 at baseline that had undergone MSC transplant and who had at least 1 year of follow-up after one or two successive intravenous injections of allogeneic BM-/UC-MSCs (1 million/kg) were analyzed. SLE symptoms and SLEDAI were assessed at baseline and during follow-up to determine low disease activity (LDA) and clinical remission (CR) at 1, 3, 6, and 12 months. Sixty-nine patients were included in the study, with a median (range) SLEDAI of 13 (8-34) at baseline. Among the 69 patients, 40 (58%) achieved LDA and 16 (23%) achieved CR with a SLEDAI of 9 (4–20), 8 (0-16), 6 (0-18), and 5 (0-18) after 1, 3, 6, and 12 months, respectively. Older age (*p* = 0.006) and no arthralgia/arthritis at baseline (*p* = 0.03) were associated with a higher rate of LDA. Achieving CR was associated with older age (*p* = 0.033), no arthralgia/arthritis at baseline (*p* = 0.001), and no prior use of cyclophosphamide (*p* = 0.003) or hydroxychloroquine (*p* = 0.016). Future studies using unique immunosuppressive regimens and allogeneic MSC sources will further elucidate determinants of clinical response to MSC transplant in SLE.

## 1. Introduction

Systemic lupus erythematosus (SLE) is a chronic multisystemic autoimmune inflammatory disorder with considerable clinical heterogeneity and a prevalence of 26 to 52 out of 100,000 [1]. Recent epidemiological studies show that the prevalence and incidence rates of SLE in Asian patients are approximately 2 to 3 times higher than those in people of European descent [2–4]. In addition, Asian SLE patients present more severe clinical manifestations, including a greater frequency of lupus nephritis, and central nervous system and cardiorespiratory involvement, all of which carry a poor prognosis when refractory to first- or second-line standard immunosuppressive drugs [5, 6]. Conventional SLE treatments, including corticosteroids, cyclophosphamide (CYC), and other immunosuppressive drugs or immunomodulating agents, can control most original flares but a definitive cure is rarely achieved [7]. Moreover, standard therapies are associated with severe side effects, including infection, ovarian failure, and secondary malignancy. Alternative therapeutic options that are more efficacious with fewer side effects are still needed to improve long-term outcome.

Ikehara et al. demonstrated in 1985 that autoimmune diseases (Ads) originate from defects in the hematopoietic system [8]. Hematopoietic stem cell (HSC) transplantation was later shown to provide a therapeutic benefit by resetting the immune system toward greater tolerance [9]. HSC transplantation is still used today for certain autoimmune diseases, although rarely in SLE [10]. The mesenchymal stem cells (MSCs) were first identified in bone marrow (BM) by Friedenstein et al. [11] as a fibroblast-like cell population capable of generating osteogenic precursors. MSCs have been extensively studied and characterized as an alternative stem cell source which have immune-modulating, immunosuppressive, and regenerative capacities [12]. Our preclinical findings from experimental animals have shown that allogeneic MSC transplantation is effective at inhibiting SLE disease progression and prolonging life in MRL/lpr mice, with improved renal function, decreased proteinuria, serum creatinine, anti-dsDNA antibodies, and reduced glomerular immune complex deposition and lymphocyte infiltration [13]. We and others later showed that allogeneic BM-MSC transplantation may be effective in human patients with refractory SLE and severe nephritis [13, 14]. Results from subsequently published trials using allogeneic BM- or umbilical cord (UC)-MSC transplants in active SLE, refractory to standard therapies [15–17], showed significant SLE remissions with lower cumulative exposure to conventional treatments.

We designed the present study to identify predictors of the 1-year clinical response to allogeneic BM- or UC-MSC transplantation from patients documented in the Nanjing cohort database.

## 2. Materials and Methods

### 2.1. Patient Eligibility

All SLE adult patients (age > 18 years at allogeneic BM-/UC-MSC treatment) from the Affiliated Drum Tower Hospital of Nanjing University Medical School database initially diagnosed with SLE by the presence of at least 4 of 11 American College of Rheumatology criteria [18] were included in the study providing they met the following inclusion criteria: (1) refractory to previous treatments, (2) a Systemic Lupus Erythematosus Disease Activity Index (SLEDAI) [19] score ≥ 8 at baseline, (3) treated by one or two intravenous injections of allogeneic BM-/UC-MSCs (1 million/kg body weight) between March 2007 and December 2013, and (4) with at least one-year follow-up after treatment. All patients provided informed consent at the time of study initiation, and the study was approved by the local Ethics Committee at the Drum Tower Hospital of Nanjing University Medical School (No. 2006006 and No. 2008017). The clinical trial registration numbers are NCT00698191 and NCT01741857.

“Refractory to previous treatment” was defined as a lack of response to monthly intravenous pulse of CYC (500-700 mg/m^2^) for ≥6 months or a lack of response to treatment with oral mycophenolate mofetil (MMF) (≥1000 mg/d) or leflunomide (LEF) (20 mg/d) for ≥3 months or continued daily doses of ≥20 mg of prednisone or its equivalent [17].

### 2.2. Allogeneic BM-/UC-MSC Preparation

Allogeneic BM-MSCs were isolated in our laboratory from bone marrow aspirates obtained from healthy donors after signing informed consent. Bone marrow mononuclear cells were separated by Ficoll density centrifugation and then cultured with low-glucose Dulbecco's modified Eagle's medium (DMEM-LG) containing 10% fetal bovine serum (FBS) and 1% penicillin-streptomycin in a humidified incubator at 37°C under 5% CO_2_.

Patients without appropriate bone marrow donors were infused with allogeneic UC-MSCs. Allogeneic UC-MSCs were prepared by the Stem Cell Center of Jiangsu Province, the National Stem Cell Institute in China. Fresh umbilical cords were obtained from healthy mothers in local maternity hospitals after normal deliveries. The cords washed in PBS with penicillin and streptomycin were then cut into 1 mm^2^ sized pieces and incubated in DMEM-LG medium containing 10% fetal bovine serum (FBS), at 37°C in humid air with 5% CO_2_.

Nonadherent cells were removed by washing. The medium was replaced every 3 days after the initial plating. When well-developed colonies of fibroblast-like cells appeared after 10 days, the cultures were trypsinized and passaged into a new flask for further expansion. At about 80–85% confluence, the adherent cells were detached by treatment with 0.125% trypsin and 0.1% EDTA.

Before infusing, cell viability was determined by trypan blue testing. The culture supernatant was also examined for microorganisms, bacteria, and fungi by direct culture analysis. In addition, supernatant virus indexes were determined by enzyme-linked immunosorbent assays. Release criteria for allogeneic BM-/UC-MSCs for clinical use included confirmation of spindle-shaped morphology and a cell viability greater than 92%, absence of visible clumps and of pathogen contamination, including screening for the following viral markers: hepatitis B surface antigen, hepatitis B core antibody, hepatitis C virus antibody, human immunodeficiency virus antibodies I and II, cytomegalovirus IgM, and syphilis antibody. Immunophenotype analysis indicated that the cultured allogeneic BM-/UC-MSCs had positive expression of CD73, CD105, CD90, and CD29 (>90%) and negative expression of CD45, CD34, CD14, CD79, and HLA-DR (<2%).

### 2.3. Allogeneic BM-/UC-MSC Transplantation

Two to four days before infusion of allogeneic BM-/UC-MSCs, patients were administered with CYC (i.v.) for the “induction phase” (10 mg per kilogram per day), unless contraindicated due to a severe baseline condition including low serum albumin (<2.5 g/dL), high serum creatinine (>3.4 mg/dL), or severe leukopenia (WBC count < 2000/*μ*L). All patients were maintained on their previous daily dose of steroid. At least one intravenous injection of 1 × 10^6^ allogeneic BM-/UC-MSCs per kilogram of body-weight was injected at baseline. A second infusion of the same dose and type of MSCs was permitted if SLE disease activity was not satisfactorily controlled or if a flare occurred within 30 days after the first injection. After allogeneic BM-/UC-MSC transplantation, the dose of prednisone was tapered by 5 to 10 mg every 2 weeks and other immunosuppressive drugs were maintained or tapered according to the SLE disease evolution as evaluated by a local rheumatologist during follow-up.

### 2.4. Patient Clinical and Biological Follow-Up and Data Analysis

SLE clinical and biological symptoms were evaluated according to the ACR classification criteria, measure of SLEDAI scores, and presence of active lupus nephritis defined by either >1.0 g per 24-hour proteinuria or ≥stage III on kidney biopsy (focal, diffuse segmental or global, membranous, and advanced sclerosing lupus nephritis) [20]. To study patients' demographics and SLE characteristics, including prior SLE medication history, specific items as described in annex 1 were extracted from the Affiliated Drum Tower Hospital of Nanjing University Medical School original database. Data were recorded at baseline (before) and at 1, 3, 6, and 12 months after allogeneic BM-/UC-MSC transplantation. During follow-up, clinical outcomes were classified according to clinical and biological SLE symptoms and the need for steroids or further immunosuppression as determined by the referring physician: (a) low disease activity (LDA) defined as SLEDAI ≤ 4 without major organ activity and prednisone ≤ 7.5 mg/day, with or without maintenance of CYC, MMF, and LEF, and (b) clinical remission (CR) defined as SLEDAI < 3 without major organ activity and prednisone ≤ 5 mg/day with or without maintenance of CYC, MMF, and LEF. In the absence of LDA, a patient was considered having “no response.”

### 2.5. Statistical Analysis

Data were analyzed using SPSS for Windows (SPSS version 22; Chicago, IL). To compare differences between the two groups, the Mann-Whitney *U* test was used to analyze continuous variables (age, SLE duration, prednisone daily dose, and SLEDAI score), expressed as the median (range), and the chi-square test to analyze categorical variables (clinical and biological symptoms, medication), expressed as percentages. The statistical significance was defined as a *p* value < 0.05.

## 3. Results

### 3.1. Patients and SLE Disease Characteristics at Baseline

Sixty-nine eligible patients (88.4%, female) were included in the present analysis.  Table 1 summarizes the SLE disease history and clinical symptoms at baseline. The median age was 32 (20-54) years, with a median SLE disease duration since diagnosis of 62 (2-264) months. The median SLEDAI score was 13 (8-34), 39.0% of patients were anti-dsDNA positive, and 43.5% had low serum complement levels. All patients had received steroids and several types of immunosuppressive therapies before treatment with allogeneic BM-/UC-MSC transplantation (Table 1).

### 3.2. Allogeneic BM-/UC-MSC Transplantation and SLE Evolution

**Figure 1:**
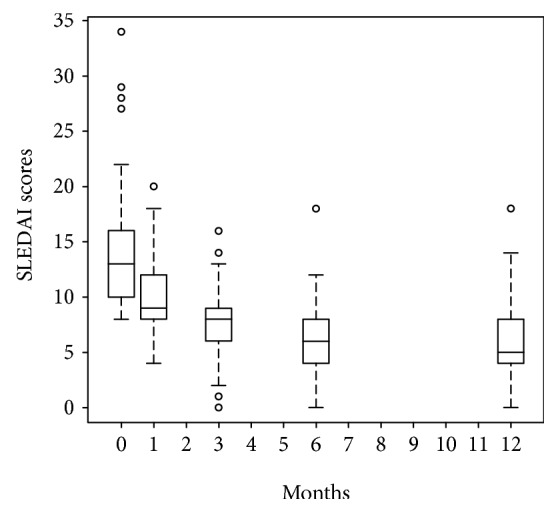
Improvement of the SLEDAI score during the 12 months following allogeneic BM/UC-MSC treatment (*p* < 0.001). During the year after allogeneic BM/UC-MSC injection, the SLEDAI score improved significantly (*p* < 0.001) over time with median values of 9 (4–20), 8 (0-16), 6 (0-18), and 5 (0-18) at 1, 3, 6, and 12 months, respectively, as compared to baseline 13 (8-34).

Between 2 and 4 days prior to allogeneic BM-/UC-MSC transplantation, 42 patients received a median CYC (i.v.) induction dose of 1.2 g (0.6-2.4). The remaining 27 patients did not receive a CYC induction due to a severe baseline condition. Eighteen patients with appropriate BM donors were treated with allogeneic BM-MSCs, and 51 patients without BM donors received an injection of allogeneic UC-MSCs. Overall, 24 out of the 69 patients received a second allogeneic BM-/UC-MSC injection within a month, using the same allogeneic MSC source and the same dose as in the first injection, with 9 patients receiving allogeneic BM-MSCs and 15 patients receiving allogeneic UC-MSCs. During the year after transplantation, the SLEDAI score improved significantly over time (*p* < 0.001; Figure 1), with median values of 9 (4–20), 8 (0-16), 6 (0-18), and 5 (0-18) at 1, 3, 6, and 12 months of follow-up, respectively. Overall, 40 (58%) patients achieved low disease activity (LDA), with 34 maintaining LDA at 12 months, and 16 (23.2%) patients achieved clinical remission (CR), with 14 maintaining CR at 12 months. 29 (42%) patients had no response during the 1-year follow-up.

### 3.3. Predictors of the Observed Clinical Response after Allogeneic BM-/UC-MSC Treatment

To identify the determinants of low disease activity (LDA) and clinical remission (CR) outcomes, we compared the distribution of clinical and biological symptoms and prior SLE medication history before allogeneic BM-/UC-MSC transplant between patients with and without LDA and CR. Older age (*p* = 0.006) and no arthralgia/arthritis at baseline (*p* = 0.03) were associated with a higher rate of LDA. All patients with neurological disorders (*n* = 5) at baseline achieved LDA.

Patients achieving CR had less frequently received CYC (*p* = 0.003) or HCQ (*p* = 0.016) prior to transplantation and were significantly older (*p* = 0.033) without arthralgia/arthritis (*p* = 0.001) at baseline, when compared to those without CR, as shown in Table 2. Overall, only 5 patients in the cohort had a neurologic disorder at entry and there was no significant difference between the 3 patients who achieved CR compared to the 2 patients who did not (*p* = 0.078). No association was found between CR and presence of anti-dsDNA, cardiorespiratory disorder, prior prednisone daily dose, CYC induction, and source of allogeneic MSCs or a second injection of allogeneic BM-/UC-MSCs.

## 4. Discussion

In the present retrospective cohort study, more than half of patients with severe refractory SLE achieved LDA in the year following transplantation of allogeneic BM-/UC-MSCs, and nearly a quarter were in CR. This is consistent with our documented observations over the last decade, since we began treating refractory SLE patients with allogenic BM-/UC-derived MSCs and suggest that transplantation of MSCs is associated with reduced disease activity, as assessed with the SLEDAI and serological markers, and improved renal function [13, 15–17]. However, there is still a need for randomized clinical trials to confirm the efficacy of allogeneic MSC transplantation in SLE [21]. Here, in collaboration with the Affiliated Drum Tower Hospital of Nanjing University Medical School and APHP Saint-Louis hospital, Paris Diderot University (No. VAL 2013/2013-076/01), we identified predictors of clinical response to help in selecting patients most likely to derive a clinical benefit from allogeneic MSC transplantation.

The strict inclusion criteria were used in this study. In order to be eligible for this study, SLE patients had to have active disease with an SLEDAI score ≥ 8 at baseline and prior successive lines of immunosuppressive drugs and steroids, with at least one-year follow-up after allogeneic MSC transplant. Our results indicate that severe SLE patients can undergo sustained clinical remission, with reduced disease activity maintained over a 1-year follow-up [17]. We identified a number of factors associated with clinical response after allogeneic MSC transplant. Older age, no arthralgia/arthritis at baseline, and no prior CYC or HCQ treatment had better first-year outcomes after allogeneic BM-/UC-MSC transplantation. Previous research has reported differences in how the disease manifests in patients with an onset after 50 years, including a lower prevalence of nephritis, CNS involvement, and dermal disturbances [22]. Formiga et al. also found that the older SLE patients have a lower SLEDAI score at diagnosis and a more benign disease course [23]. In addition, lower SLE disease activity was observed by Bertoli et al. [24] in patients with late disease onset. Although arthralgia/arthritis is a very common manifestation in SLE, observed in 90% of patient's irrespective of disease activity stage, it is less frequently observed when SLE disease onset occurs at an older age [22]. In the present study, although all SLE patients were refractory to standard treatments, those who did not have a prior medication history that included CYC or HCQ had a better treatment response after allogeneic BM-/UC-MSC transplantation. Interestingly, when the efficacy of belimumab (anti-BLyS) has been demonstrated, it was shown that patients with SLEDAI above 8 were more sensitive to treatment, despite being refractory to previous standard therapies [25]. Together, our results indicate that clinical symptoms and medication history at baseline can influence the response to allogeneic BM-/UC-MSC transplantation.

There were several limitations in this retrospective cohort study. A relatively small number of patients met our strict inclusion criteria, resulting in a small sample size. However, these inclusion criteria were necessary in order to inform decisions regarding patient selection in future randomized, double-blind, and multicenter-controlled studies assessing allogeneic MSC transplantation in SLE. Further research with a larger sample size is needed. Second, it can be argued that immunosuppressive medications used as “induction” therapy together with sustained steroids contributed to the results observed during follow-up after allogeneic BM-/UC-MSC injection. However, all subjects included were refractory to all previous therapies, and disease activity indeed decreased after allogeneic BM-/UC-MSC transplantation. Third, this single-center study only included patients of Chinese ethnicity, and therefore, the results may not be extrapolated to SLE patients of other ethnic origins.

## 5. Conclusions

In conclusion, allogeneic BM-/UC-MSCs may be efficacious in treating patients with active disease who are refractory to standard treatments, with our results projecting potential rates of LDA around 58% and CR around 23% in the year following transplantation. Future studies using unique immunosuppressive regimens and allogeneic MSC sources in collaboration with the EBMT will further elucidate the determinants of clinical response to allogeneic MSCs, a promising therapeutic option in multisystemic SLE.

## Figures and Tables

**Table 1:** Patient's characteristics at baseline (*n* = 69 patients).

Variables	*n* = 69
Prior SLE medication history	
Prednisone use	69 (100%)
Prednisone daily dose/mg, median (range)	20 (5-500)
CYC use	47 (68.1%)
MMF use	19 (27.5%)
HCQ use	31 (44.9%)
LEF use	14 (20.3%)
Clinical and biological symptoms	
Fever	25 (36.2%)
Rash	44 (63.8%)
Photosensitivity	33 (47.8%)
Oral ulcers	12 (17.4%)
Arthralgia/arthritis	49 (71.0%)
Serositis	17 (24.6%)
Vasculitis	14 (20.3%)
Alopecia	19 (27.5%)
Renal disorder	64 (92.8%)
Active lupus nephritis	63 (91.3%)
Neurologic disorder	5 (7.3%)
Cardiorespiratory disorder	19 (27.5%)
Hematological disorder	32 (46.4%)
Immunologic disorder	40 (58.0%)
Antinuclear antibodies	54 (78.3%)
Anti-dsDNA	27 (39.1%)
Anti-Sm	21 (30.4%)
Anti-SSA	17 (24.6%)
Anti-SSB	6 (8.7%)
Low complement	30 (43.5%)
CYC induction	42 (60.9%)
Allo-MSC transplantation	
Sources of MSCs (BM/UC)	18/51
Second MSCs	24 (34.8%)
SLEDAI score at baseline, median (range)	13 (8-34)
CR during 1 year	16 (23.2%)
LDA during 1 year	40 (58.0%)

SLE: systemic lupus erythematosus; Anti-dsDNA: anti-double-stranded DNA antibody; Allo-MSCs: allogenic mesenchymal stem cells; BM: bone marrow-derived; UC: umbilical cord-derived; SLEDAI: Systemic Lupus Erythematosus Disease Activity Index; CYC: cyclophosphamide; MMF: mycophenolate mofetil; HCQ: hydroxychloroquine; LEF: leflunomide; CR: clinical remission; LDA: low disease activity.

**Table 2:** Patient's characteristics divided by CR.

Variables	CR (*n* = 16)	Without CR (*n* = 53)	*p* value
Age/years, median (range)	38 (23-54)	30 (20-54)	0.03
SLE duration/months, median (range)	53.5 (13-232)	78 (2-264)	0.33
Female	15 (93.75%)	46 (86.79%)	0.67^∗^
Prior SLE medication history			
Prednisone daily dose (mg)			
≤20	5 (31.25%)	23 (43.4%)	0.39
>20	11 (68.75%)	30 (56.6%)	
CYC use	6 (37.5%)	41 (77.4%)	0.003
MMF use	4 (25.0%)	15 (28.3%)	0.80
HCQ use	3 (18.8%)	28 (52.8%)	0.02
LEF use	1 (6.3%)	13 (24.5%)	0.11
Clinical and biological symptoms			
Fever	5 (31.3%)	20 (37.7%)	0.64
Rash	12 (75.0%)	32 (60.4%)	0.38^∗^
Photosensitivity	10 (62.5%)	23 (43.4%)	0.18
Oral ulcers	4 (25.0%)	8 (15.1%)	0.45^∗^
Arthralgia/arthritis	6 (37.5%)	43 (81.1%)	0.001
Serositis	1 (6.3%)	16 (30.2%)	0.09^∗^
Vasculitis	5 (31.3%)	9 (17.0%)	0.21
Alopecia	5 (31.3%)	14 (26.4%)	0.70
Renal disorder	14 (87.5%)	50 (94.3%)	0.33^∗^
Active lupus nephritis	13 (81.3%)	50 (94.3%)	0.10
Stage ≥ 3 lupus nephritis®			
Stage III	0 (0%)	2 (20%)	0.63
Stage IV	1 (100%)	5 (50%)	
Stage V	0 (0%)	3 (30%)	
Neurologic disorder	3 (18.8%)	2 (3.8%)	0.08^∗^
Cardiorespiratory disorder	2 (12.5%)	17 (32.1%)	0.20^∗^
Hematological disorder	7 (43.8%)	25 (47.2%)	0.81
Immunologic disorder	8 (50.0%)	32 (60.4%)	0.46
Antinuclear antibodies	11 (68.8%)	43 (81.1%)	0.29
Anti-dsDNA	6 (37.5%)	21 (39.6%)	0.88
Anti-Sm	4 (25.0%)	17 (32.1%)	0.76^∗^
Anti-SSA	6 (37.5%)	11 (20.8%)	0.17
Anti-SSB	0 (0%)	6 (11.3%)	0.32^∗^
Low complement	7 (43.8%)	23 (43.4%)	0.98
CYC induction			
CYC induction	7 (43.8%)	35 (66.0%)	0.11
<1.2 g	0 (0%)	3 (8.6%)	0.42
≥1.2 g	7 (100%)	32 (91.4%)	
Allo-MSC transplantation			
UC-MSCs	10 (62.5%)	41 (77.4%)	0.33
Second MSCs	4 (25.0%)	20 (37.7%)	0.35
SLEDAI median (range)			
Baseline	13 (9-34)	13 (8-27)	0.72
M1	6 (4-20)	10 (4-18)	0.01
M3	6 (0-16)	8 (4-14)	0.04
M6	4 (0-8)	7 (3-18)	<0.001
M12	2 (0-8)	6 (4-18)	<0.001

CR: clinical remission; SLE: systemic lupus erythematosus; Anti-dsDNA: anti-double-stranded DNA antibody; Allo-MSCs: allogenic mesenchymal stem cells; BM: bone marrow-derived; UC: umbilical cord-derived; SLEDAI: Systemic Lupus Erythematosus Disease Activity Index; CYC: cyclophosphamide; MMF: mycophenolate mofetil; HCQ: hydroxychloroquine; LEF: leflunomide; M: follow-up months. ^∗^Fisher test. ®Only for the patients with available kidney biopsy.

## Data Availability

The data used to support the findings of this study are available from the corresponding author upon request.
